# Supplementary material for: Highly stretchable polymer semiconductor thin films with multi-modal energy dissipation and high relative stretchability
Source: Nat Commun. 2023 Dec 16;14:8382. doi: 10.1038/s41467-023-44099-w (PMC10725446; doi:10.1038/s41467-023-44099-w)
Supplement: Supplementary file 1 — Supplementary information [file 41467_2023_44099_MOESM1_ESM.pdf]

Supplementary Information

Highly stretchable polymer semiconductor thin films with multi-modal energy dissipation and high relative stretchability

Hung-Chin Wu,[1] Shayla Nikzad,[1] Chenxin Zhu,[2] Hongping Yan,[1,3] Yang Li,[4] Weijun Niu,[4] James R. Matthews,[4] Jie Xu,[1,#] Naoji Matsuhisa,[1,$] Prajwal Kammardi Arunachala,[5] Reza Rastak,[5] Christian Linder,[5] Yu-Qing Zheng,[1] Michael F. Toney,[3,&] Mingqian He,[4,] Zhenan Bao[1,*]*

[1]Department of Chemical Engineering, Stanford University, Stanford, CA 94305, United States

[2]Department of Electrical Engineering, Stanford University, Stanford, CA 94305, United States

[3]Stanford Synchrotron Radiation Lightsource, SLAC National Accelerator Laboratory, Menlo Park, CA 94025, United States

[4]Corning Incorporated, Corning, New York 14831, United States

[5]Department of Civil and Environmental Engineering, Stanford University, Stanford, CA 94305, United States

[#]Present address: Nanoscience and Technology Division, Argonne National Laboratory, Lemont, IL 60439, United States

[$]Present address: Institute of Industrial Science, The University of Tokyo, Meguro, Tokyo 153-8505, Japan

[&] Present address: Department of Chemical and Biological Engineering and Renewable and Sustainable Energy Institute (RASEI), University of Colorado Boulder, Boulder, CO 80309, United States

Corresponding author. Email: hem@corning.com (M.H.); zbao@stanford.edu (Z.B.)

Materials

P2TBDPP2TBFT4, with molecular weights from approximately 20 to 100 kg mol^{-1} (P1-P4) were synthesized according to reported methods.[S1] Poly(dimethylsiloxane) (PDMS, Sylgard 184, Dow Corning) was prepared at a ratio of 12:1 (base:cross-linker, w/w) and cured at 70 °C for 1 hour for usage in the film transfer and as the substrate of PSC stretching characterizations. Octadecyltrimethoxylsilane (OTS) and all the solvents, such as chlorobenzene and trichloroethylene, were purchased from Sigma-Aldrich and used as received.

Supplementary Table S1 | Electrical performance of FETs based on biaxially stretched P4 films with uneven strains

Biaxial strain	Electrode direction vs. strain direction[a]	Mobility[b] ($cm^2V^{-1}s^{-1}$)	On/off ratio[b]	Threshold voltage[b] (V)	On-current[b] (A)
25×50%	parallel	0.211±0.004	2×10^4	-1.6±0.3	$(2.7\pm0.07)\times10^{-5}$
	//	0.195±0.019	5×10^3	0.3±0.2	$(2.5\pm0.26)\times10^{-5}$
	perpendicular	0.176±0.022	1×10^4	-0.8±0.7	$(2.1\pm0.08)\times10^{-5}$
	direct average between parallel and perpendicular[c]	0.194	2×10^4	-0.4	2.4×10^{-5}
50×75%	parallel	0.168±0.035	3×10^4	-1.3±0.8	$(1.9\pm0.34)\times10^{-5}$
	//	0.162±0.011	2×10^4	-0.6±0.4	$(1.7\pm0.25)\times10^{-5}$
	perpendicular	0.131±0.007	2×10^4	-1.9±0.9	$(1.5\pm0.07)\times10^{-5}$
	direct average between parallel and perpendicular[c]	0.149	3×10^4	-1.6	1.7×10^{-5}
50×100%	parallel	0.239±0.032	4×10^4	0.3±0.4	$(2.7\pm0.29)\times10^{-5}$
	//	0.184±0.016	3×10^4	1.1±0.9	$(2.0\pm0.05)\times10^{-5}$
	perpendicular	0.108±0.013	1×10^4	-1.8±0.2	$(1.3\pm0.13)\times10^{-5}$
	direct average between parallel and perpendicular[c]	0.174	2×10^4	-0.8	2.0×10^{-5}

[a]The parallel direction of electrode means the charge transport direction is parallel to the direction of the larger strain direction. The perpendicular direction of electrode means the charge transport direction is the same as the direction of the smaller strain. Additionally, charge transport was probed with the channel rotated 45° with respect to either parallel or perpendicular direction (labeled as //). As a result, slightly higher mobility is shown at the direction with higher strain, which is due to the polymer chain alignment.

[b]The electrical performances are averaged from at least 5 devices from 2 different batches.

[c]The direct average of electrical behaviors between parallel and perpendicular directions show that the 45° performance is generally the same as the average of the parallel and perpendicular, especially with respect to the FET on-current.

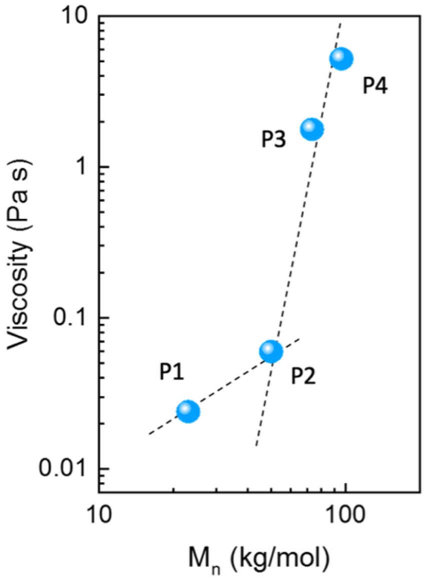

Supplementary Figure S1 | Solution viscosity of P1-P4 measured using a rheometer. The polymer solutions were prepared in chlorobenzene at a concentration of 1 mg mL^{-1} and the rotation speed during the measurement was fixed as 10 rad s^{-1}. The experiment was conducted at 25 °C. As the molecular weight increased, the solution viscosity became greater. Note that the increment of viscosity is significantly larger when the molecular weight is larger than approximately 50 kg mol^{-1}, indicating the formation of polymer chain entanglements. One set of samples was measured.

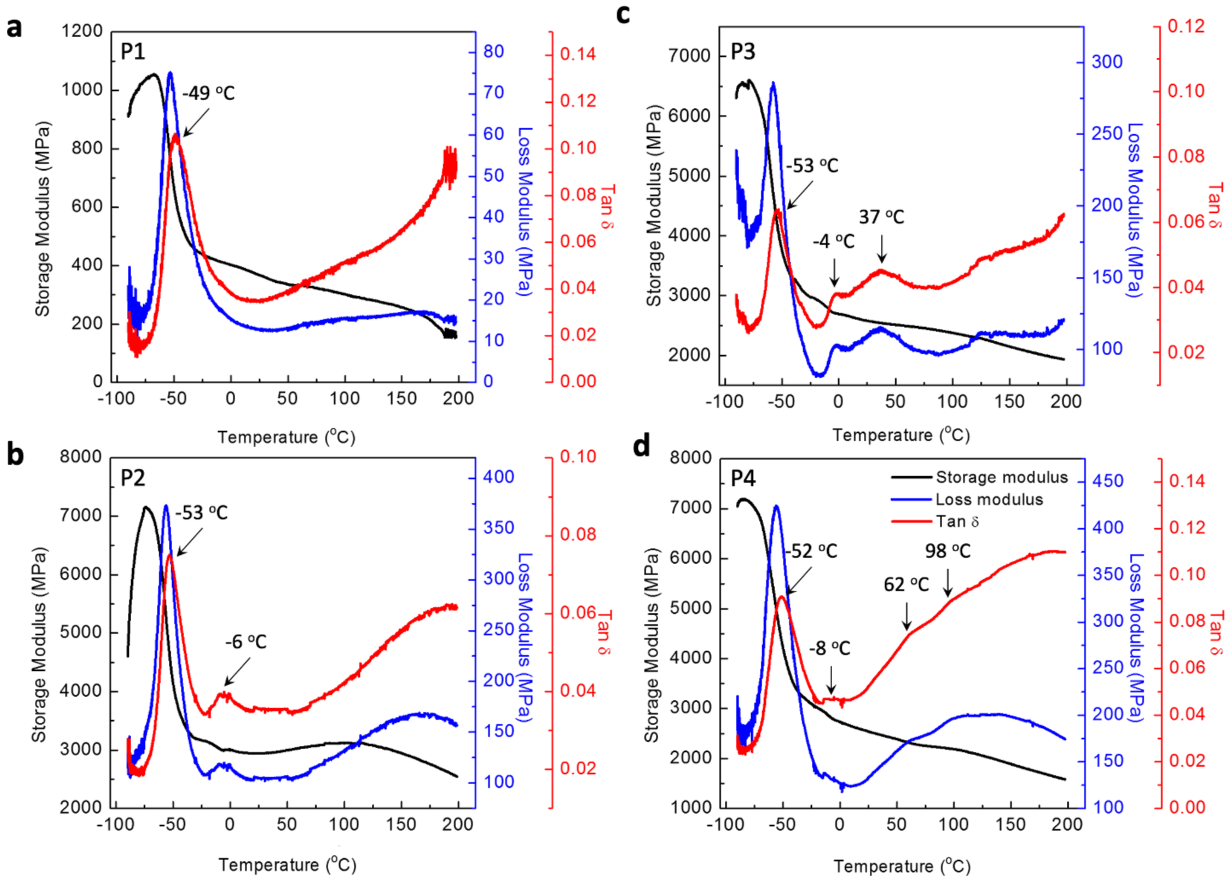

Supplementary Figure S2 | Molecular dynamics of bulk polymer films. Dynamic mechanical analysis of (a) P1, (b) P2, (c) P3, and (d) P4 samples (> 1 μm-thick film) measured from -90 to 200 °C. Relatively rigid P1 and P2 showed only glass transition temperatures (T_g) of the alkyl side chain at approximately -50 and -5 °C,[S2] while P3 and P4 showed not only side chain T_g but also backbone T_g located above room temperature, implying the polymer chains in high molecular weight polymers have more free volumes. 2 samples were measured with similar thermal behaviors. The representative plots are shown.

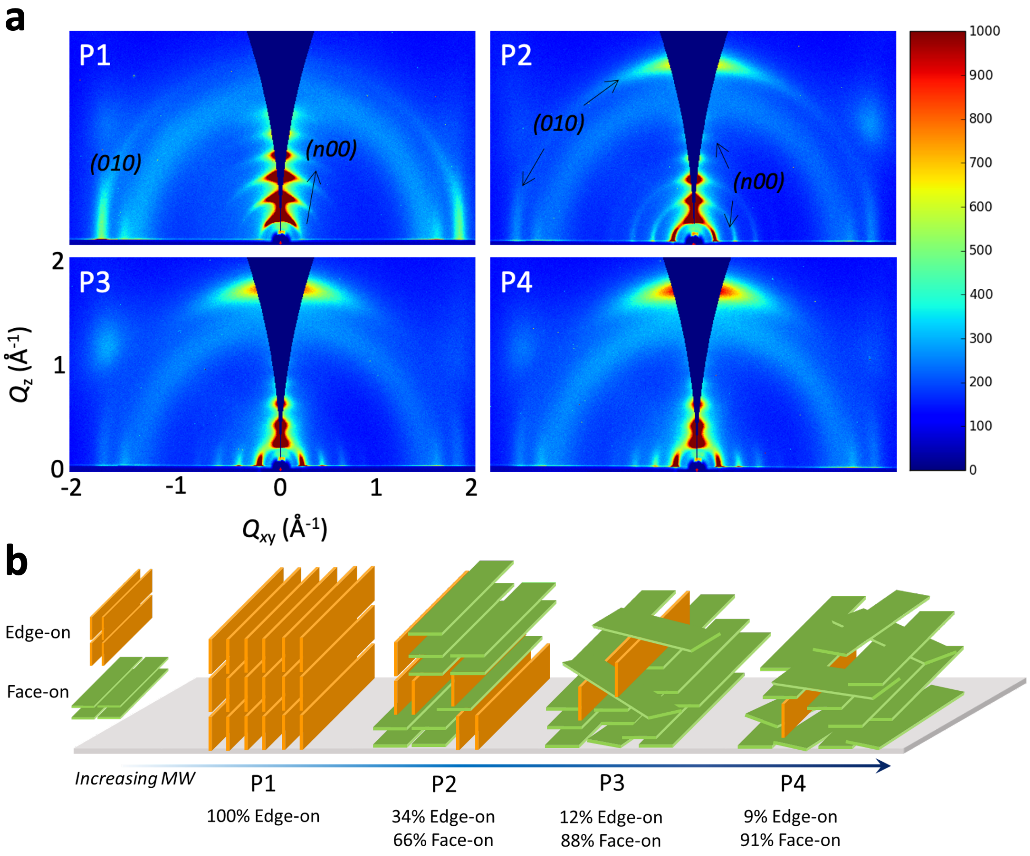

Supplementary Figure S3 | Orientations of crystalline domains in polymer thin films (~ 50 nm). (a) 2D GIXD patterns and (b) the illustration of the corresponding molecular packing features of P1-P4 thin films. P1 exhibited lamella *(n00)* and π-π staking *(010)* peaks in Q_z and Q_{xy} directions, respectively, indicating edge-on packing. P2-P4 exhibited both edge-on and face-on packing orientations in thin film state as indicated by both *(n00)* and *(010)* reflections precent in both Q_z and Q_{xy} directions at a same time. Significant differences in crystalline packing in polymer films can be observed for polymers of different molecular weights. 3 samples were measured for each polymer, and similar trends were observed. The representative patterns are shown.

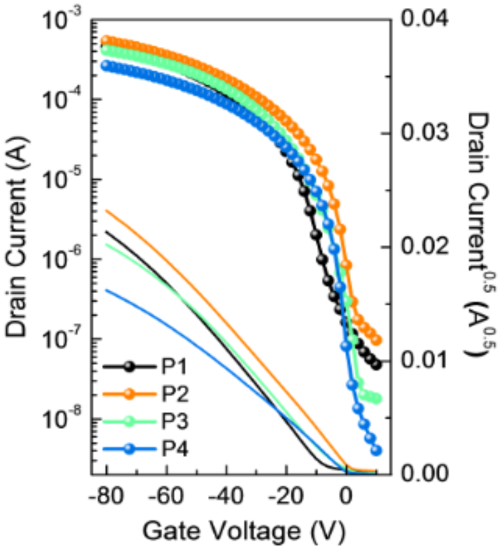

Supplementary Figure S4 | Charge transport properties of polymer thin films. Transfer characteristics of FET devices. Since the dimensions of the devices are identical, a higher on-state current indicates a grater charge carrier mobility. P2 shows a higher field-effect transistor (FET) mobility, which may be due to its balanced edge-on and face-on packing orientation facilitating 3D charge transport. The source-to-drain voltage was set as -100V, channel length and width are 50 and 1000 μm, respectively, and the dielectric layer is 300 nm-thick SiO_2 with a capacitance of 10.9 nF cm^{-2}. 10 devices from 2 batches were measured, and they showed similar trends. The representative plots are shown.

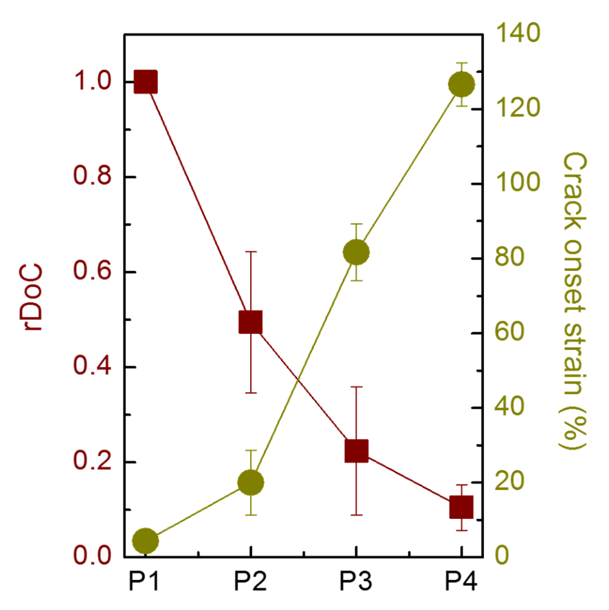

Supplementary Figure S5 | Crystallinity and crack onset strain of polymer thin films. The relative degree of crystallinity (rDoC) and crack onset strain of P1-P4 thin films (~ 50 nm). The molecular ordering was directly impacted by molecular weight, which in turn greatly impacts the thin film mechanical properties. The relative degree of crystallinity (rDoC) was extracted from the *(200)* diffraction peak in the GIXD pattern. At least 2 samples over 2 batches were measured.

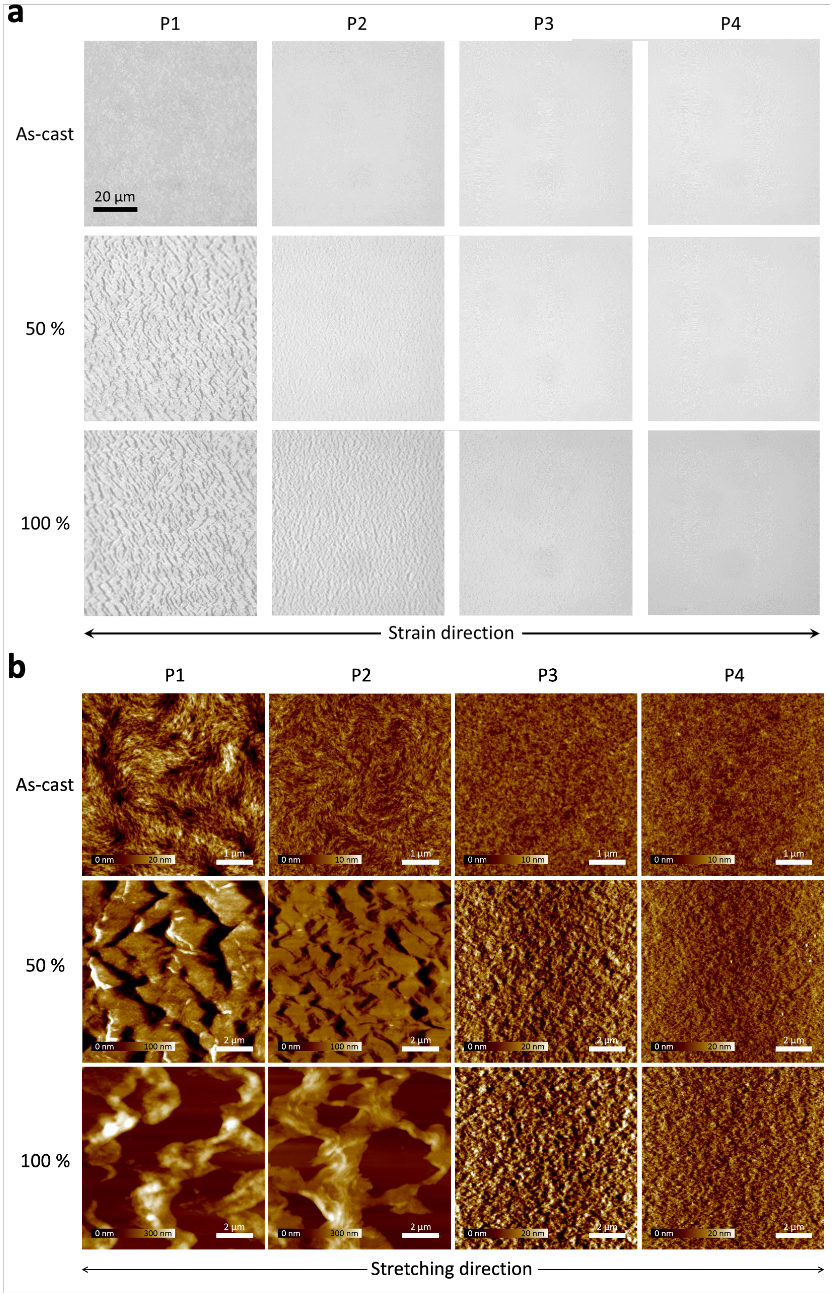

Supplementary Figure S6 | Characterization of the mechanical stretchability and surface morphology. (a) Optical microscopy images and (b) atomic force microscopy (AFM) topographies of P1-P4 films under various strains. Thin films with higher molecular weight exhibit much higher tolerance against external strains. It is clearly shown that lower molecular weight polymer form more brittle films (i.e. P1 and P2) and show more and larger cracks on the surface under strain. Smooth and continuous films were observed for the high molecular weight films (i.e. P3 and P4).

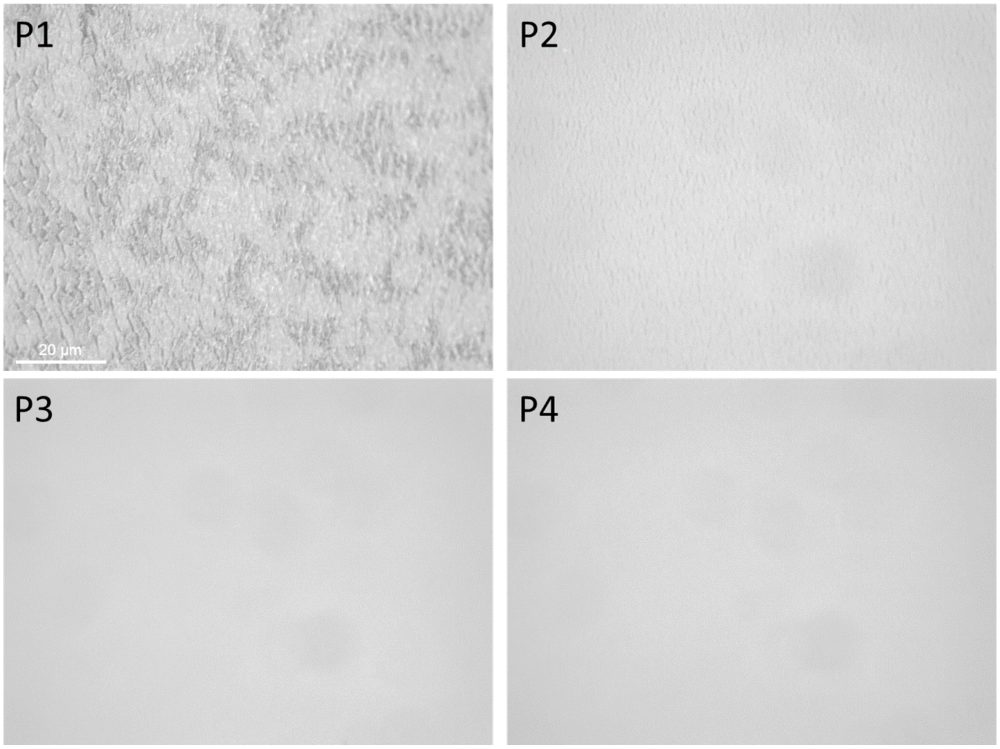

Supplementary Figure S7 | Optical microscopy images of strain-released P1-P4 films. The thin films (approximately 50 nm) supported on PDMS substrates were strained to 100% strain and then released for these microscope images. Damages can be observed on lower molecular weight and low *rS* value P1 and P2 films, while P3 and P4 films stayed smooth and continuous without noticeable defects.

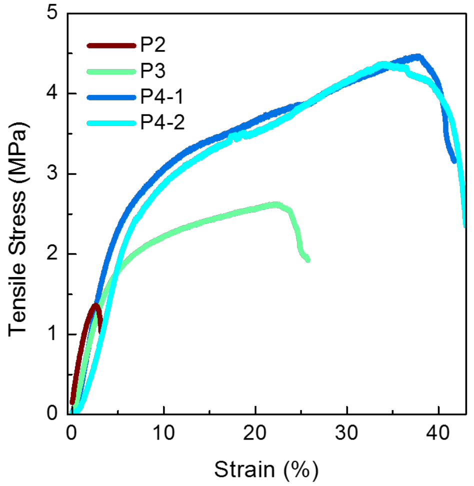

Supplementary Figure S8 | Mechanical properties of polymer bulk films. Stress-strain curves of bulk, free-standing P2, P3, and P4 thick films (~2-3 μm-thick). Polymers with a higher molecular weight showed a higher stretchability (P2, P3, and P4 samples broke at 2, 23, and 37%, respectively). 1 sample was measured on P2 and P3, and 2 samples were measured on P4.

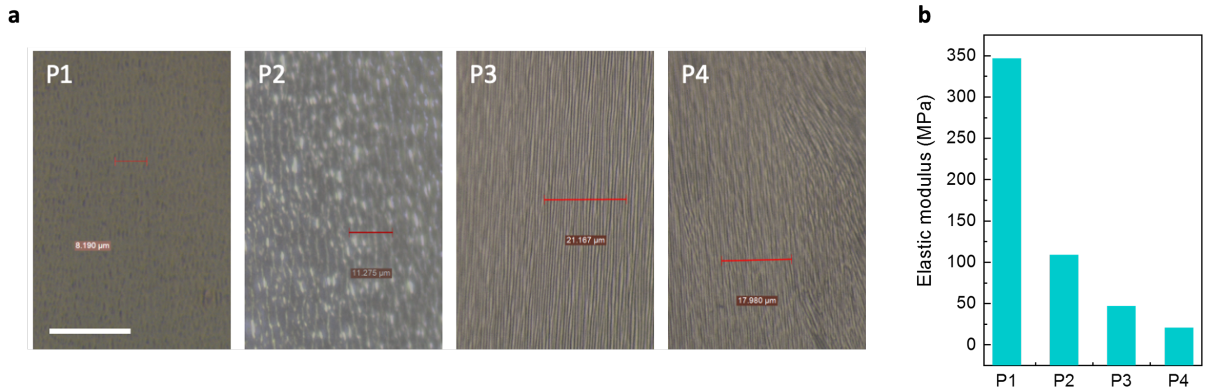

Supplementary Figure S9 | Modulus measurements of P1-P4 thin films. (a) The buckling method was used to determine the elastic modulus of polymer thin films, and the buckling features were captured using optical microscopy. The averaged wavelength of buckles and film thickness are 1.49 μm and 50 nm for P1, 1.61 μm and 58 nm for P2, 0.81 μm and 50 nm for P3, and 0.62 μm and 47 nm for P4, respectively. The scale bar in the optical microscopy image is 20 μm. (b) Calculated elastic modulus of P1-P4 films using the reported method[S3] with an average of 3 samples.

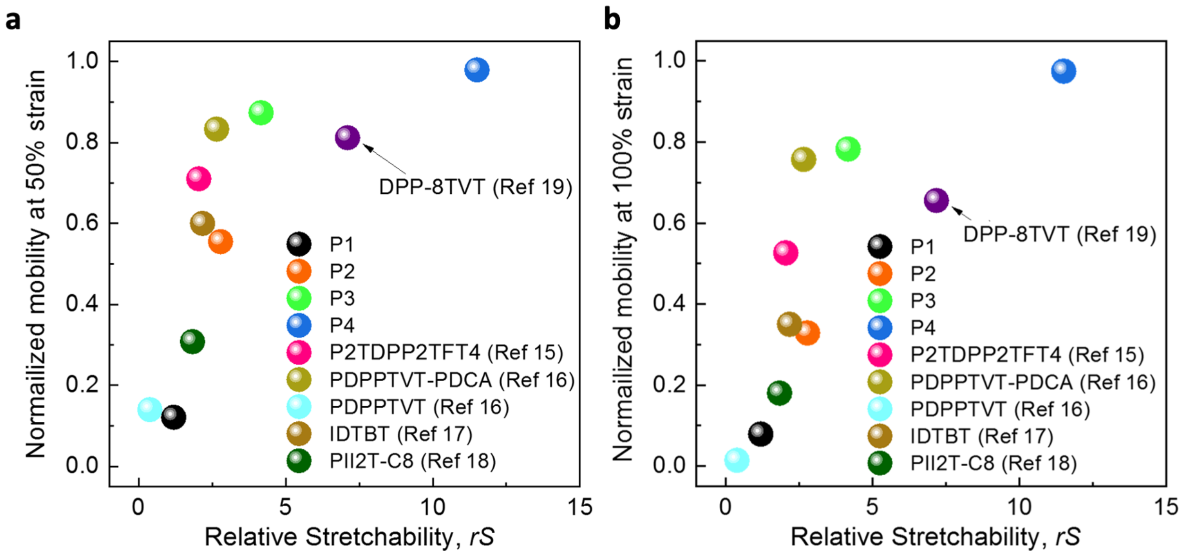

Supplementary Figure S10 | Correlations between charge carrier mobility and *rS* values of terpolymer DPP-8TVT. Normalized FET mobility under (a) 50 and (b) 100% uniaxial strain (i.e. mobility under 50 or 100% strain divided by the mobility at 0% strain) of the studied and reported PSCs compared with terpolymer DPP-8TVT. Normalized mobility for P1-P4 were averaged from at least 5 devices over 2 batches. Results from literatures were extracted from corresponding papers based on reported data.

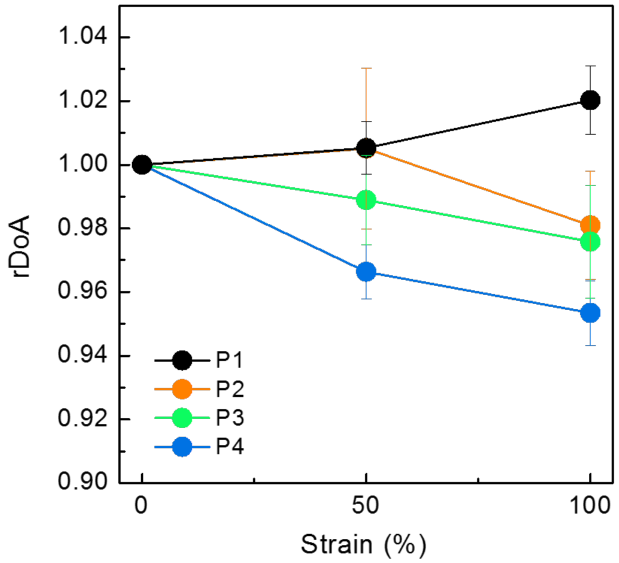

Supplementary Figure S11 | Degree of polymer aggregation in P1-P4 thin films under strain. Relative degree of aggregation (rDoA) of P1-P4 films under strain was extracted from the ratio of (0-0) and (0-1) peaks in UV-vis spectra by 3 different samples. The rDoAs of low molecular weight P1 and P2 polymers showed relatively lower changes of rDoA under strain because the polymer films were damaged at low strain levels. On the contrary, the high molecular weight P3 and P4 exhibited continuous and larger rDoA decrease due to the continuous deformation of the polymer films.

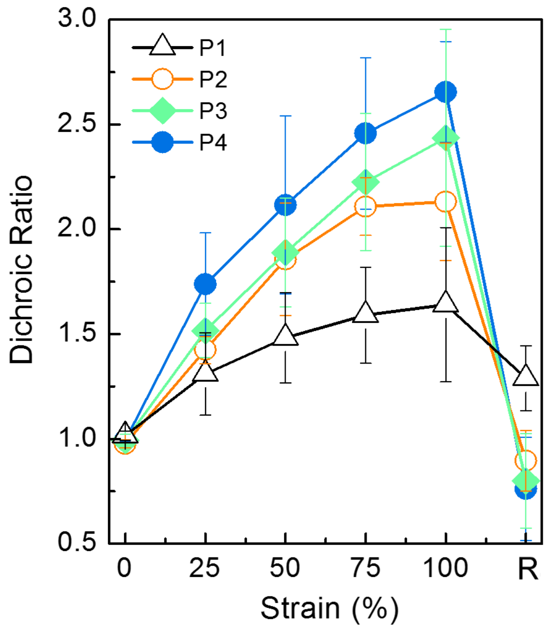

Supplementary Figure S12 | Polymer chain alignment of P1-P4 thin films under strain. Dichroic ratio (DR) of P1-P4 films under strain. A higher degree of polymer chain alignment is indicated by a larger DR, implying better ability for polymer chains to undergo extension and alignment when an external strain is applied. It should be noted that the DR value of the P1 film stayed above 1 while P2-P4 films dropped to below 1 when the applied strain was released (i.e. 100 to 0% strain; R%), indicating there were chain re-orientations. DR values are averaged from 3 samples each.

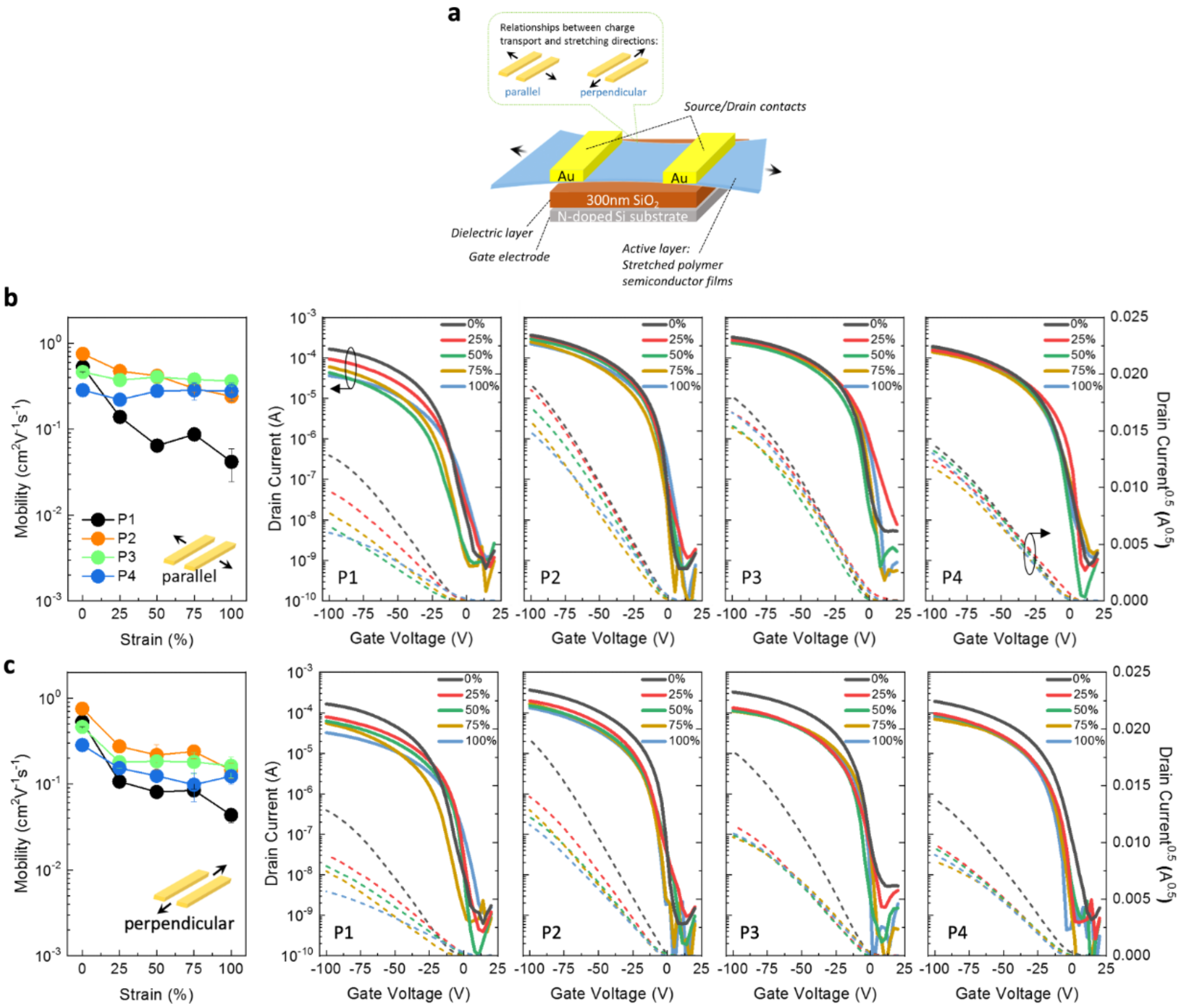

Supplementary Figure S13 | Electrical properties of stretched P1-P4 thin films. (a) Schematic structures of bottom-gate/top-contact FET devices. Stretched PSC films were transferred onto the Si/SiO_2 substrate, and the source and drain contacts were deposited parallel or perpendicularly to the stretching directions. Note that only the PSC layer was stretched to extract the relationship between charge carrier mobility and applied strain in order to avoid potential damage by strain at the interface between PSC and the electrodes or dielectric layer. Averaged charge carrier mobility (for 10 devices from at least 2 batches) and representative transfer curves of P1-P4-based FETs under strain with the charge transport direction defined (b) parallel or (c) perpendicularly to the stretching direction, respectively. Although polymers with lower molecular weights (P1 and P2) exhibited higher initial mobilities (i.e. non-stretched state), their FET performance decayed significantly once the films were deformed. On the contrary, the higher molecular weight polymers (P3 and P4) maintained charge transport under 100% strain. The source-to-drain current was set as -100V, channel length and width are 50 and 1000 μm, respectively, and the dielectric layer is 300 nm-thick SiO_2 with a capacitance of 10.9 nF cm^{-2}.

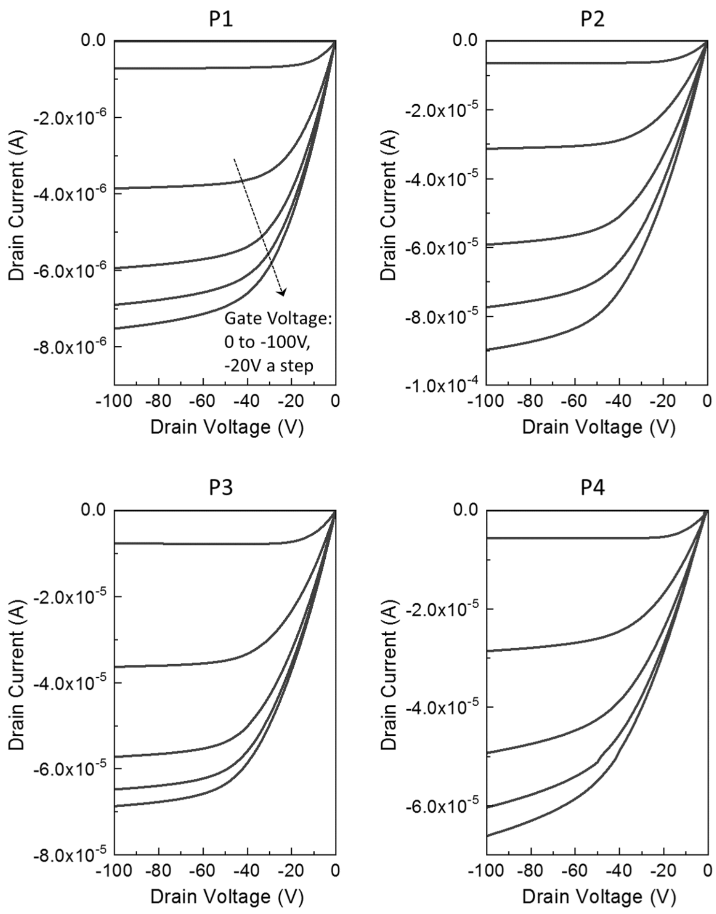

Supplementary Figure S14 | Representative FET output characteristics of P1-P4-based devices under 100% strain. The channel length and width are 50 and 1000 µm, respectively, and the dielectric layer is 300 nm-thick SiO_2 with a capacitance of 10.9 nF cm^{-2}.

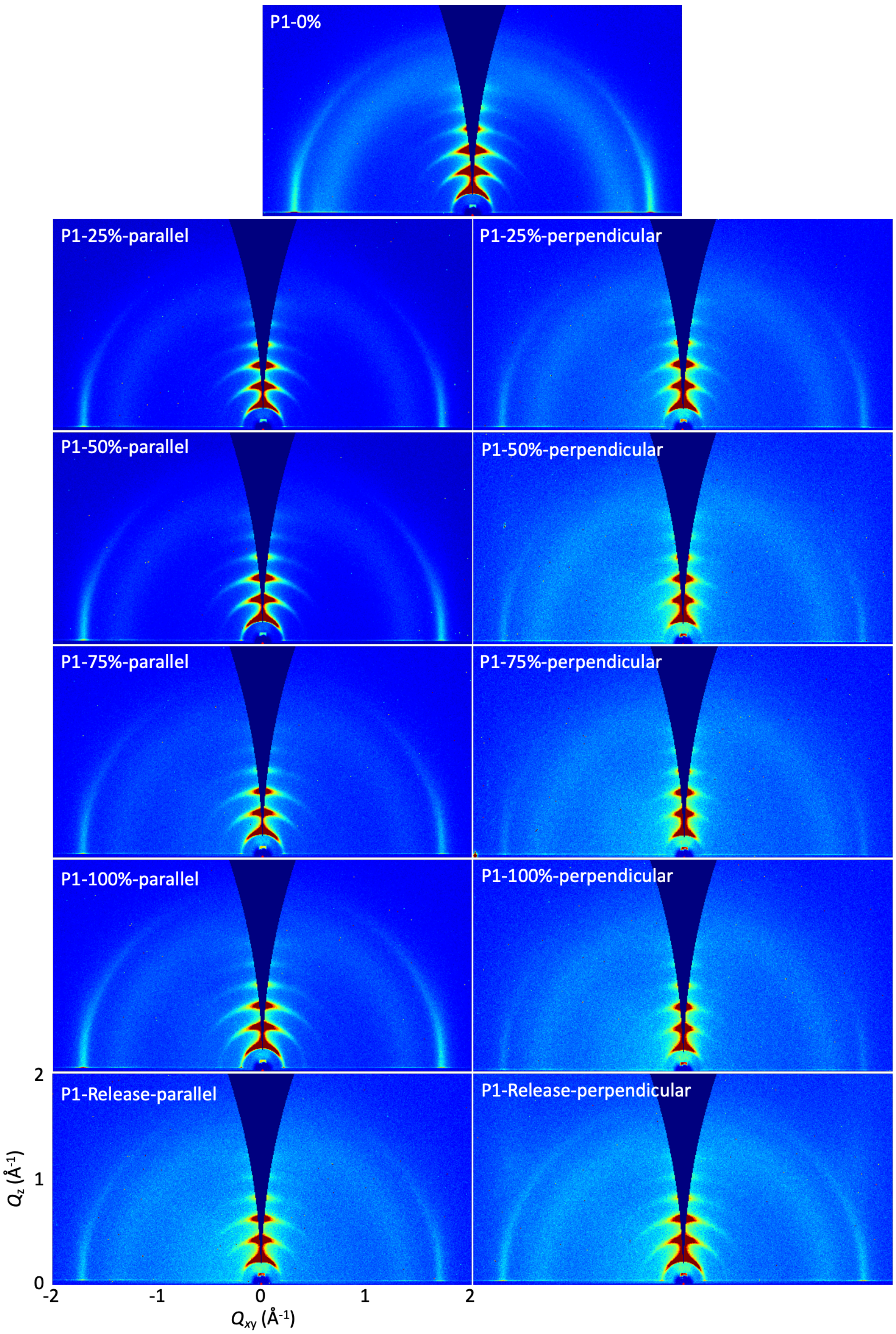

Supplementary Figure S15 | 2D GIXD patterns of P1-based films under various strain levels. The incident X-ray beam was controlled to be parallel or perpendicular with respect to the stretching direction. 3 samples from 3 different batches were measured and they showed similar trends. The representative plots are shown.

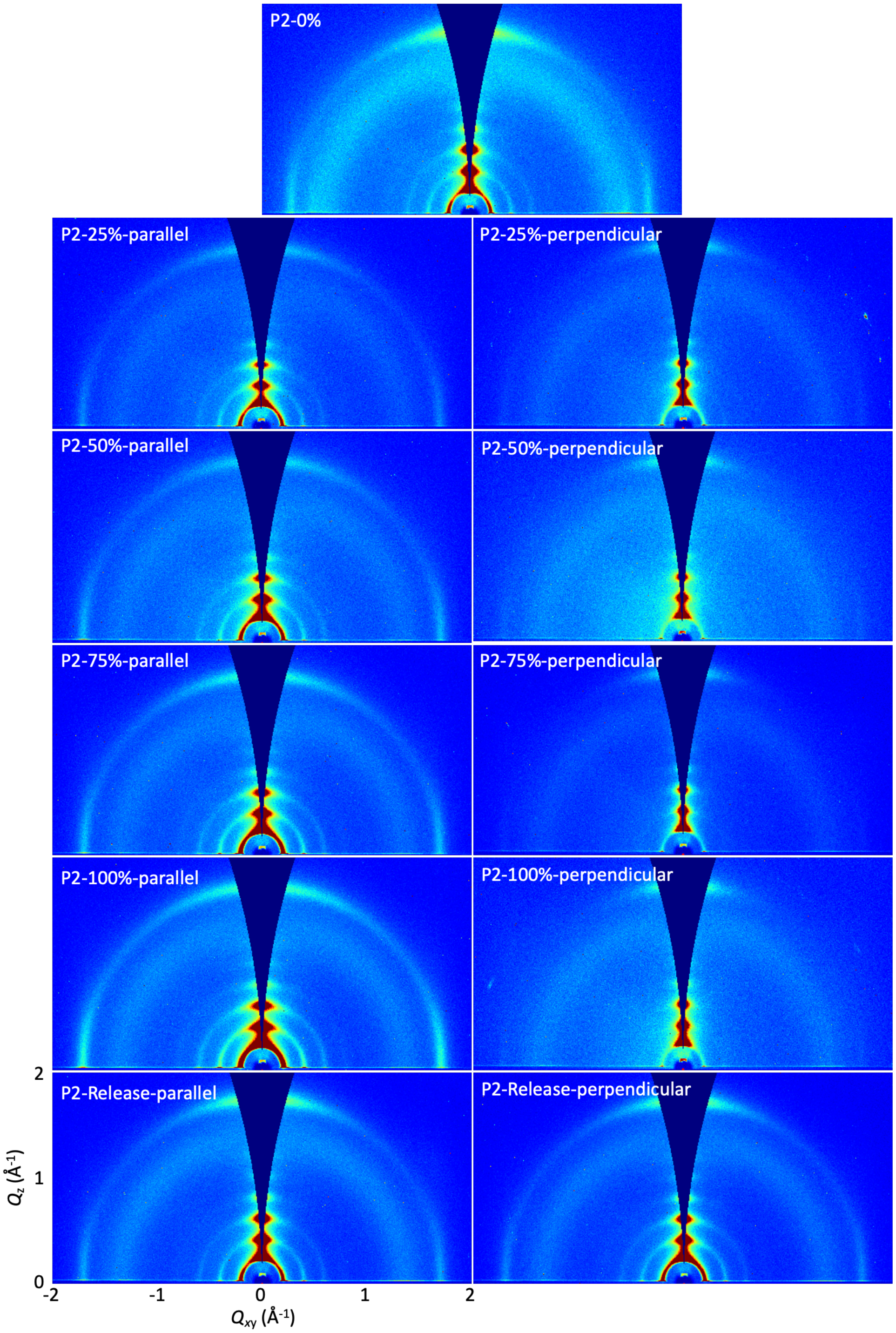

Supplementary Figure S16 | 2D GIXD patterns of P2-based films under various strain levels. The incident X-ray beam was controlled to be parallel or perpendicular with respect to the stretching direction. 3 samples from 3 different batches were measured and they showed similar trends. The representative plots are shown.

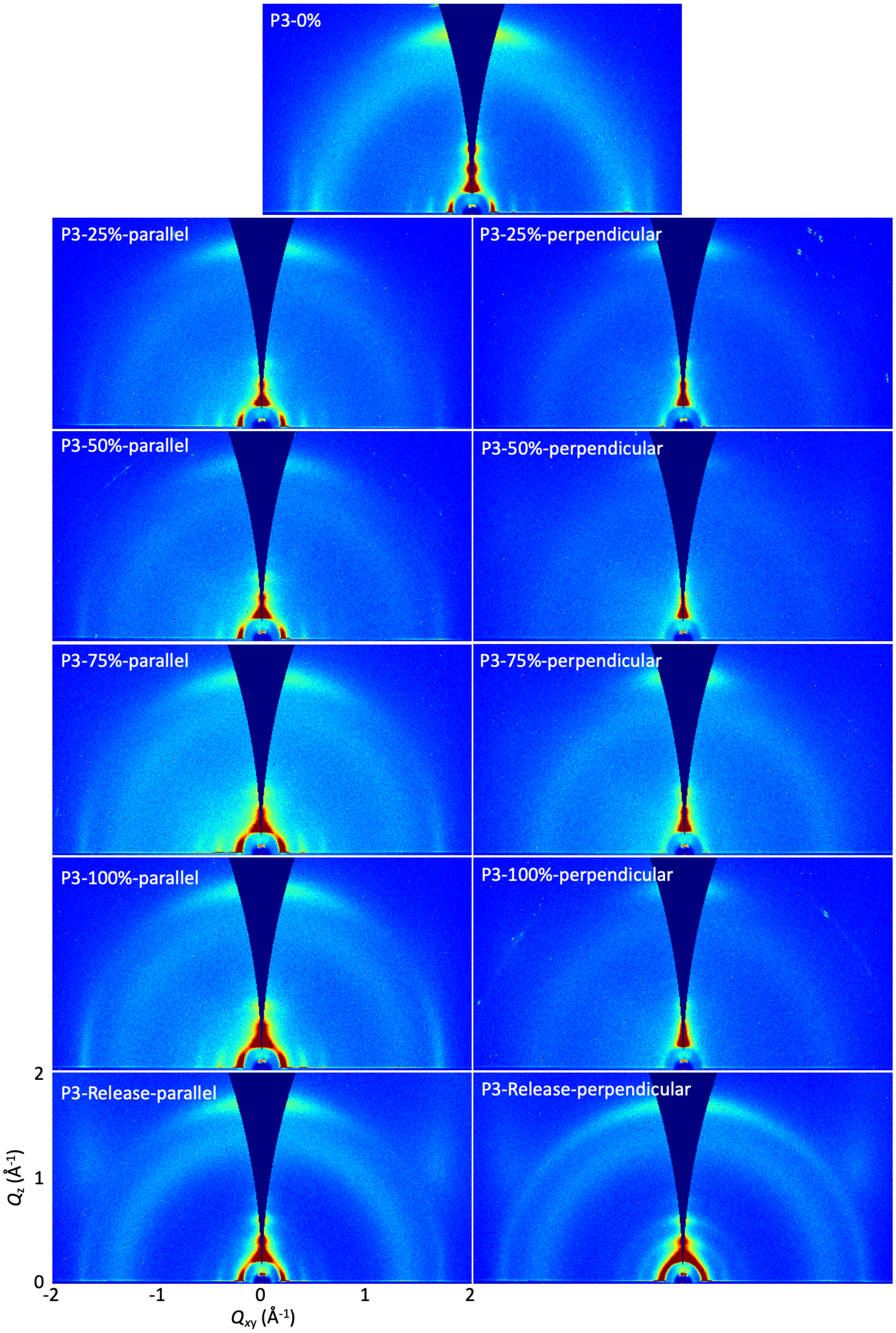

Supplementary Figure S17 | 2D GIXD patterns of P3-based films under various strain levels. The incident X-ray beam was controlled to be parallel or perpendicular with respect to the stretching direction. 3 samples from 3 different batches were measured and they showed similar trends. The representative plots are shown.

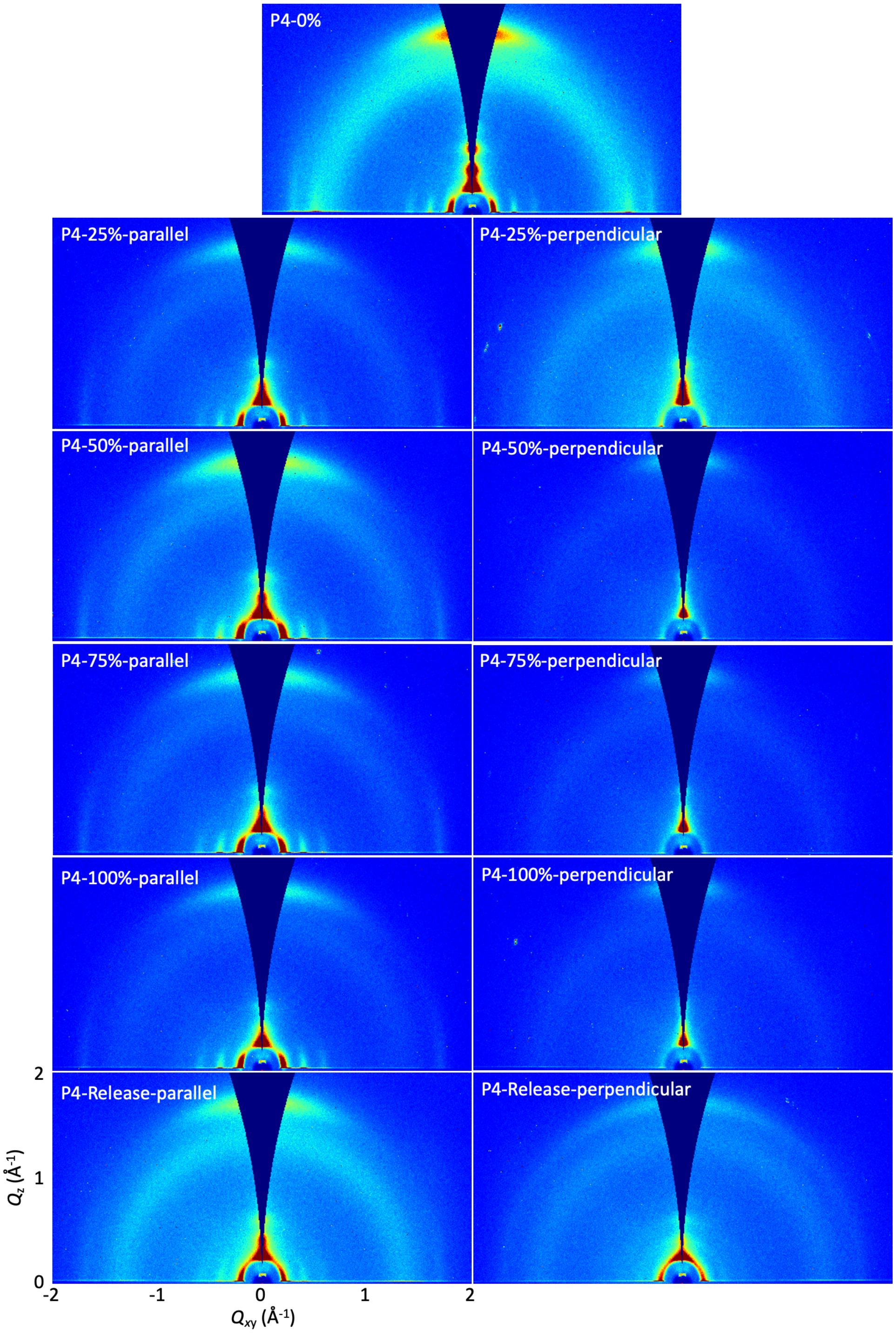

Supplementary Figure S18 | 2D GIXD patterns of P4-based films under various strain levels. The incident X-ray beam was controlled to be parallel or perpendicular with respect to the stretching direction. 3 samples from 3 different batches were measured and they showed similar trends. The representative plots are shown.

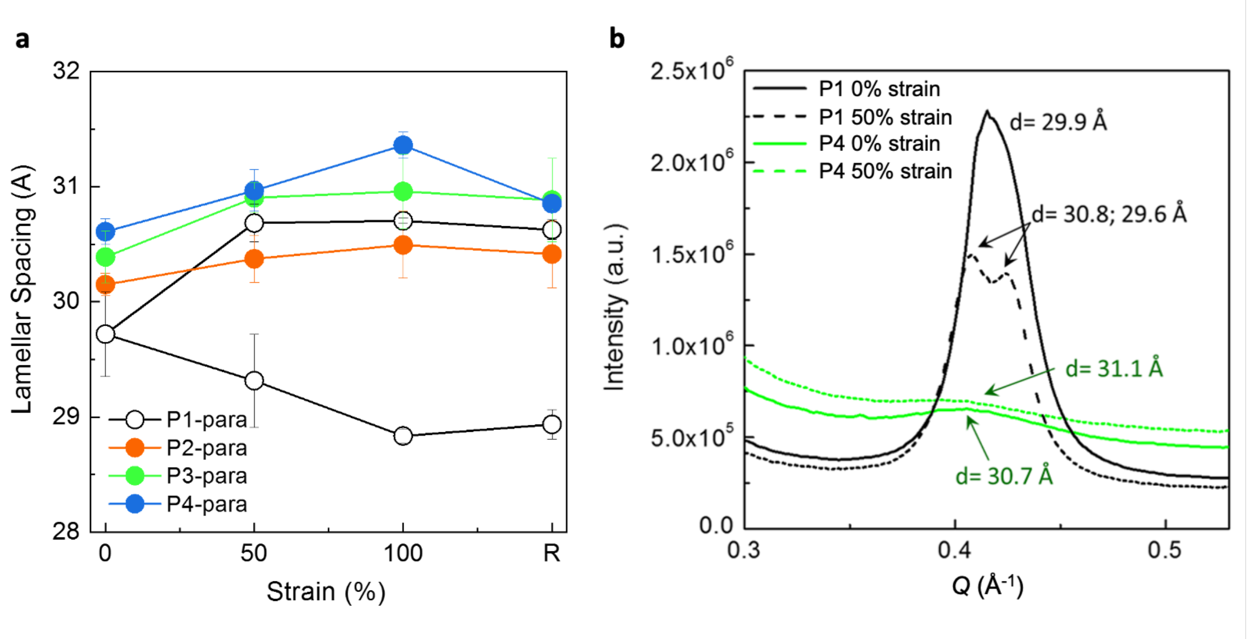

Supplementary Figure S19 | Molecular packing structure of crystalline domains inside polymer films under strain. (a) Averaged lamellar spacing of P1-P4 films as a function of strain for 2-3 samples over 2 batches. The spacing distance is calculated from *(200)* peak from the 2D GIXD pattern when the incidence X-ray is set parallel to the stretching direction. (b) 1D GIXD profiles of *(200)* peak (integrating all signals in both Q_{xy} and Q_z directions) of P1 and P4 films under 0 and 50% strain. The most brittle P1 film among the studied polymers showed two distinct lamellar spacings after an external strain was applied, indicating the crystalline domains were disrupted during stretching or new polymorphs were formed. On the contrary, P2-P4 films presented single spacings under strain and the spacing was enlarged when the strain level increased, which suggests that the ordered domains were deformed but not fractured by the external force. More interestingly, when the films were released from 100 back to 0% strain (i.e. R%), the spacing of P4 films returned back to its initial (non-stretch state; 0%) spacing, indicating that the crystalline domains were not permanently damaged under strain. The spacing of P2 and P3 films at R% strain, however, stayed the same as the films under strain, implying that the applied forces affected the molecular ordering permanently.

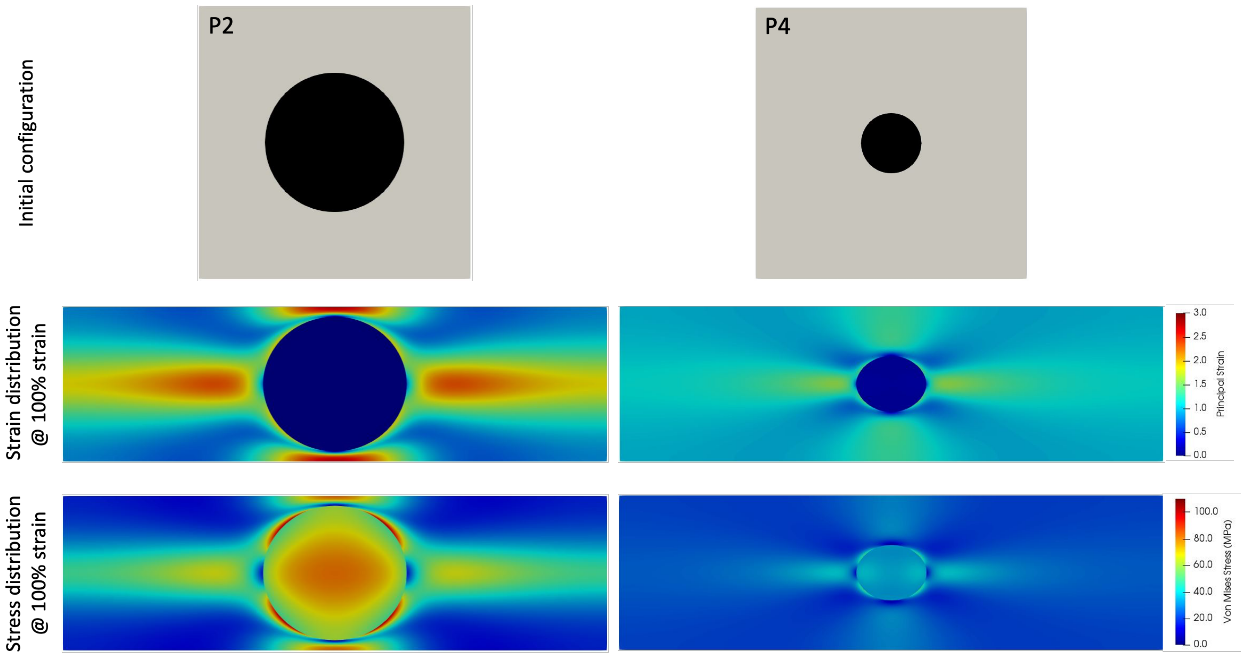

Supplementary Figure S20 | Mechanical strain and stress distribution on polymer films under uniaxial stretch. A 2D simulation of a square film with a circular inclusion under a uniaxial global engineering strain of 100% is performed to explore the mechanical stability of the system under large stretches. Assuming a uniform distribution of the crystallites inside the amorphous domain, a part of the domain with a single crystallite is modeled. The length of the initial (unstretched) square film is taken as 90 nm, and the size of the inclusion is defined as 23 nm and 10 nm for P2 and P4, respectively, which is the mean crystallite size calculated from GIXD results. The Neo-Hookean large deformation hyper-elastic material is utilized for modeling the materials. The elastic modulus of the inclusion (i.e. crystalline domain) is set to 2000 MPa for P2 and 200 MPa for P4 and the modulus of the film (i.e. amorphous domain) is assumed to be 20 MPa. Note that the values of the moduli are estimated from the morphological and mechanical characteristics of polymer films. A highly refined mesh with around 760,000 and 240,000 elements for P2 and P4 films respectively is considered with a biased refinement at the junction of the crystalline-amorphous regions. A symmetric boundary condition is considered by assuming the lateral boundaries to be flat in order to emulate the practical behavior of a single system within a large amorphous domain containing many crystallites. The distributions of the principal strains and von-Mises stresses (which are a measure of the maximum stresses) are investigated for studying the mechanical stability against the failure of the system. Significantly different stress and strain distributions are observed for the P2 and P4 films. The low molecular weight P2 film has a larger crystalline domain and thus it accumulates a large maximum stress of 90.1 MPa on the crystalline domain and 104.4 MPa on the film, while the high molecular weight P4 film exhibits a smaller maximum stress of 47.4 MPa on the crystalline domain and 48.6 MPa on the film. The strain field

shows a considerable jump along the interface between the amorphous and the crystalline regions in both P2 and P4 simulations, caused by the large difference in the elastic stiffnesses of the two regions. Also, the P2 film shows the concentration of high strains as large as 270% on the amorphous domain while the P4 film has a relatively smaller 158% strain concentration effected due to the stretch. These simulations clearly indicate the enhanced mechanical stability of the P4 films as compared to the P2 films.

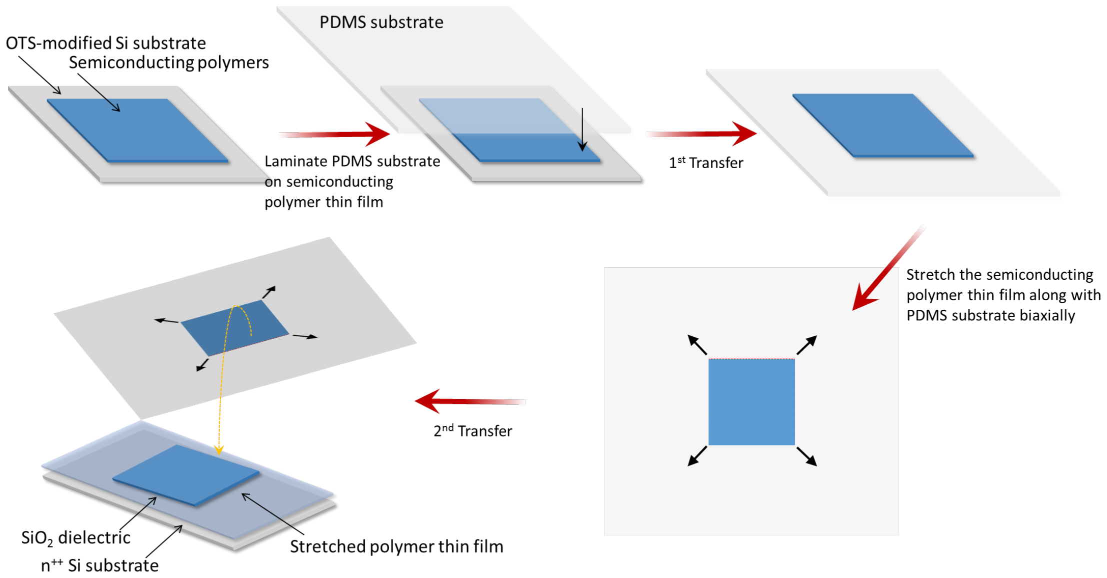

Supplementary Figure S21 | Illustration of biaxial stretching of PSC thin films. The biaxially stretched polymer films were prepared using the transfer method. The PSC films were first spin-coated on the OTS-modified Si substrate and then transferred onto a PDMS substrate. Afterwards, the PSC films were stretched biaxially along with the PDMS substrate. Finally, the biaxially stretched PSC films were transferred back to Si substrate for device or morphological measurements.

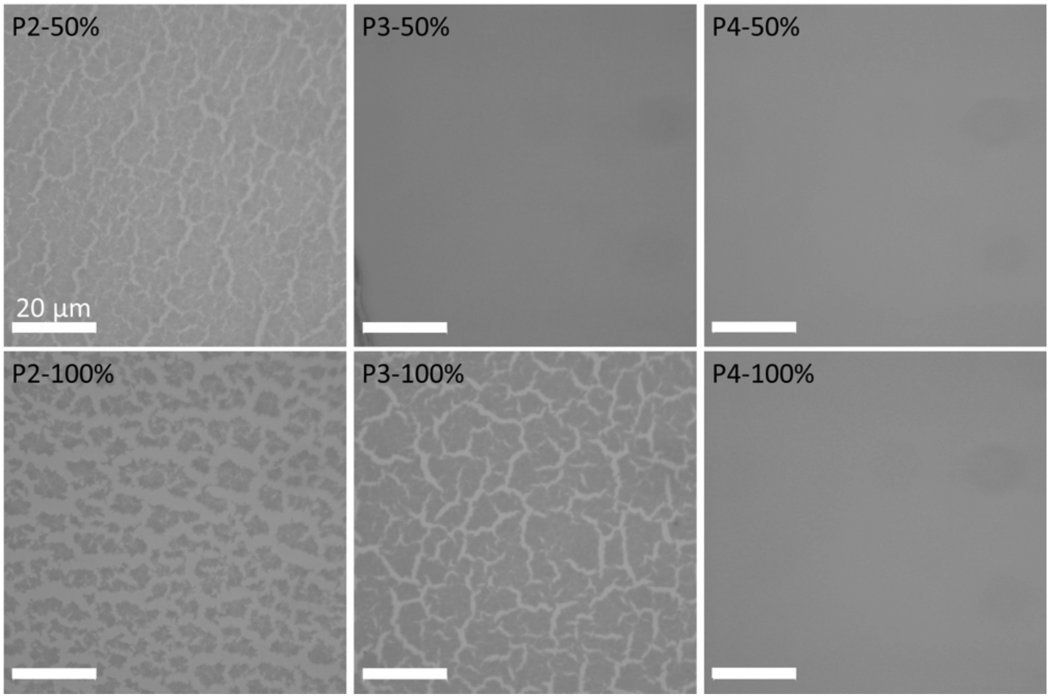

Supplementary Figure S22 | Characterization of the mechanical stretchability under biaxial strains. Optical microscopy images of P2, P3, and P4 thin film under 50 and 100% biaxial strain (225 and 400% aerial expansion). Micron-scale cracks are formed on P2 and P3 films after a certain force is applied, while a smooth anon-cracked surface is observed on the P4 film, leading to stable electrical performance under biaxial strain.

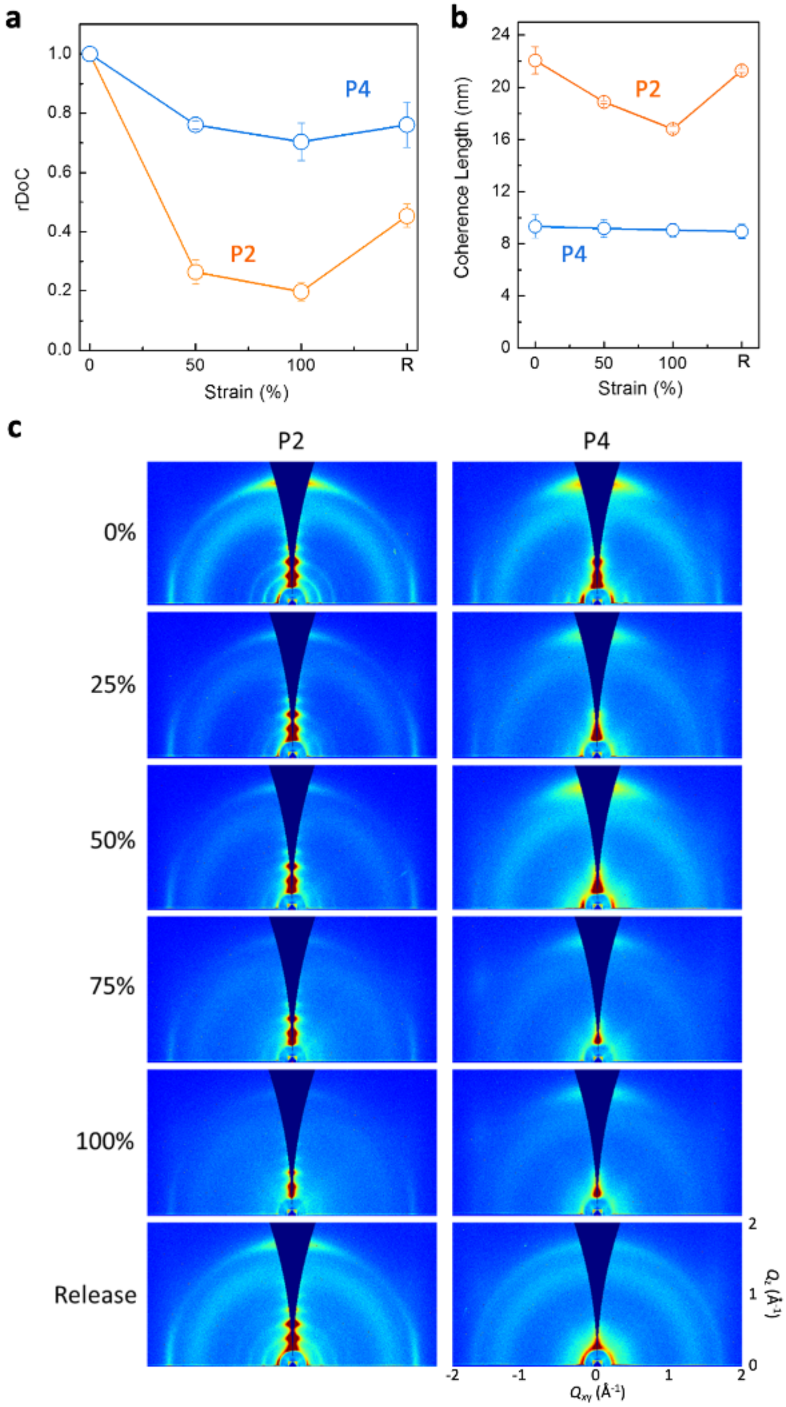

Supplementary Figure S23 | Molecular organizations under biaxial strain. Molecular organization of crystalline domains, including averaged (a) rDoC and (b) coherence length values of P2 and P4 films under various biaxial strain levels. The ductile P4 film can maintain its crystalline structure under deformation, leading to stable electrical performance. (c) The corresponding 2D GIXD patterns of P2 and P4 films. 2 samples over 2 batches were measured for each polymer, and they showed similar trends. The representative plots are shown.

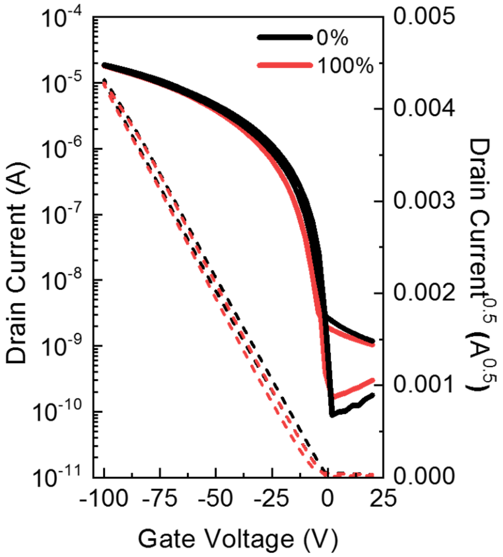

Supplementary Figure S24 | Representative dual-sweep transfer characteristics of P4-based FETs under 0 and 100% biaxial strain. Comparable electrical behaviors and negligible hysteresis were exhibited by the P4 active layer under 0 (i.e. non-stretched) and 100% biaxial strain. 2 FET devices from 2 different batches were measured, and they showed similar trends. The source-to-drain current was set as -100V, channel length and width are 150 and 1000 μm, respectively, and the dielectric layer is 300 nm-thick SiO_2 with a capacitance of 10.9 nF cm^{-2}.

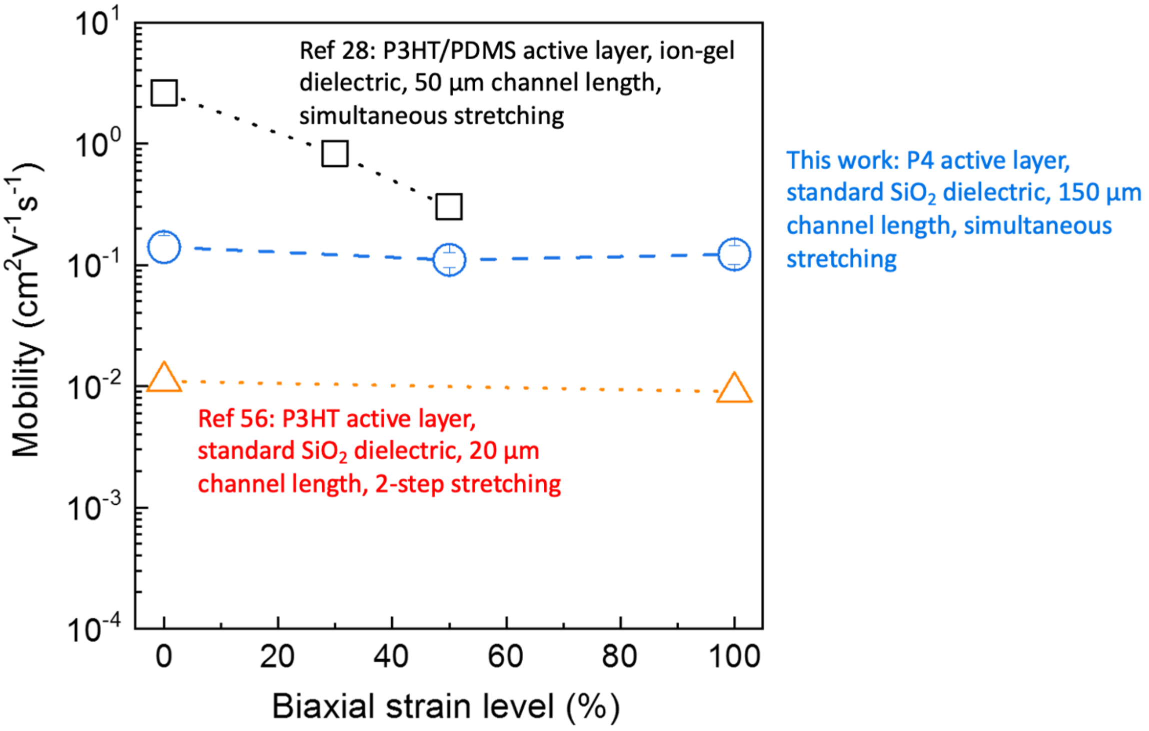

Supplementary Figure S25 | PSC-based FET devices under biaxial strain. Summarized charge carrier mobilities of FETs based on PSC under biaxial strain. At least 5 P4-based devices in this study over 2 batches were measured and those results from literature were extracted from corresponding papers based on reported data. Until now, only 2 previous studies reported results from operation of FET devices under biaxial strain. In ref 56, P3HT was stretched one direction to 100% and then stretched the orthogonal direction (2-step) to realize 100% biaxial strain. Although the mobility was not significantly degraded after stretching, the FET performance was generally poor (i.e. with a mobility of ~0.01 $cm^2V^{-1}s^{-1}$); on the other hand, P3HT/PDMS blend (ref 28) could be stretched simultaneously to biaxial 50% strain with the help of an elastomeric material (i.e. PDMS). However, the mobility decayed dramatically (over 85%) after stretching. Note that the mobility in that study is relatively higher than common P3HT-based FETs due to the usage of an ion-gel dielectric layer. Compared to those pioneer works, our PSC (i.e. P4) can achieve a mobility higher than 0.1 $cm^2V^{-1}s^{-1}$ under simultaneous biaxial strain up to 100% without adding elastomeric components. In addition, the electrical performance stays stable before and after stretching. Indeed, P4 in this study has demonstrated a record high performance for a PSC-based FET device under multidirectional strain, which is important for real-world soft electronic applications.

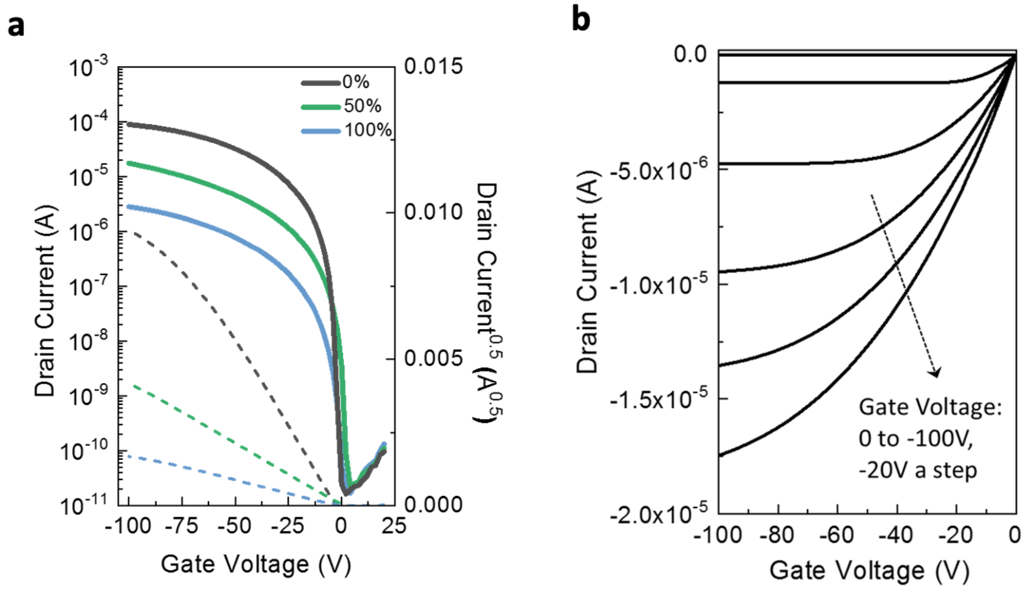

Supplementary Figure S26 | Electrical performance under 100% biaxial strain. (a) Transfer curves of P3 thin film under biaxial strain. The source-to-drain current was set as -100V, channel length and width are 150 and 1000 µm, respectively, and the dielectric layer is 300 nm-thick SiO_2 with a capacitance of 10.9 nF cm^{-2}. Similar to P2-based devices, decay of electrical properties is observed with increasing strain. (b) FET output characteristics of P4 film under 100% biaxial strain. At least 5 FETs over 2 batches were measured, and the representative plots are depicted.

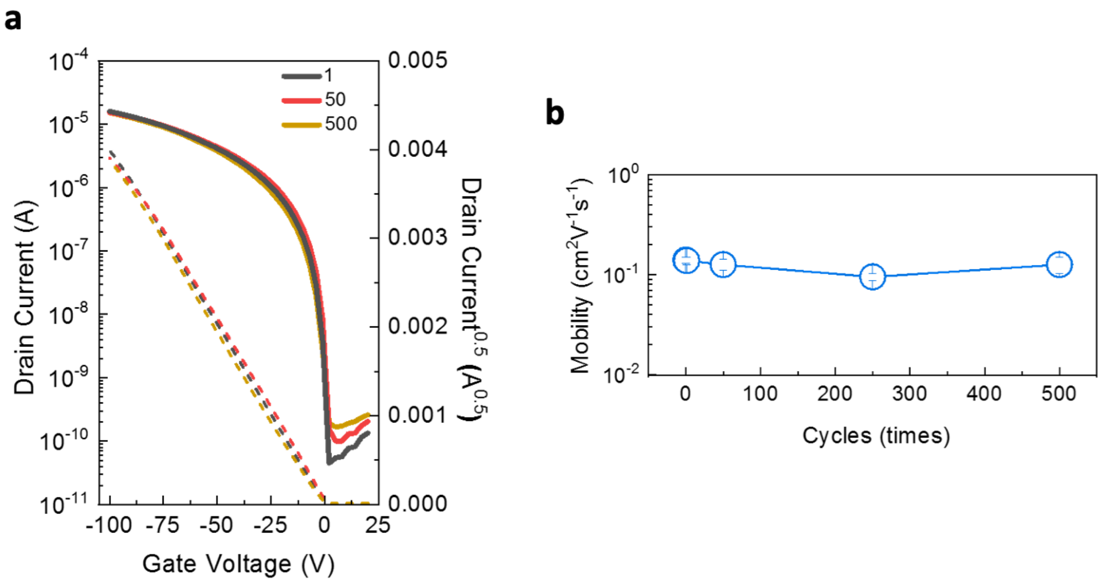

Supplementary Figure S27 | Operational durability of P4 film under 100% biaxial strain. The P4-based FET devices were fabricated from active layers exposed to 1, 50, 250, and 500 stretching/releasing cycles. The corresponding (a) transfer curves and (b) charge carrier mobility are depicted. Excellent operational stability was exhibited as the charge carrier mobility remained above 0.1 $cm^2V^{-1}s^{-1}$ throughout the cycling process. The source-to-drain current was set as -100V, channel length and width are 150 and 1000 µm, respectively, and the dielectric layer is 300 nm-thick SiO_2 with a capacitance of 10.9 nF cm^{-2}. The FET mobility was averaged from 3 samples over 2 batches.

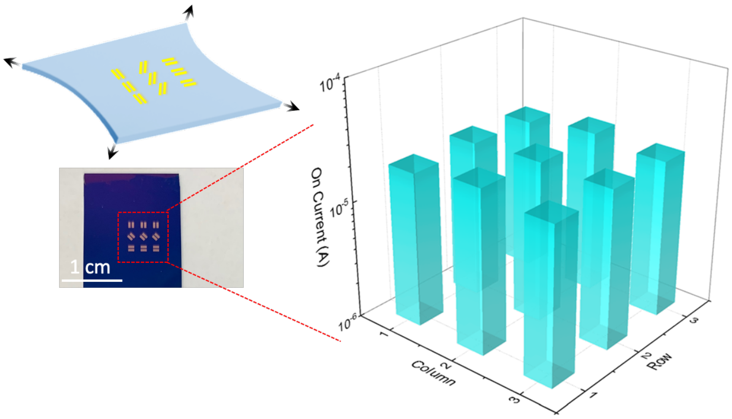

Supplementary Figure S28 | Electrical performance of the P4 film under 100% biaxial strain. Different charge transport directions with respect to strain direction were investigated. The on-current is not affected by the stretching direction, indicating isotropic charge transport in the biaxially deformed films. The source-to-drain current of FET devices was set as -100V, channel length and width are 150 and 1000 μm, respectively, and the dielectric layer is 300 nm-thick SiO_2 with a capacitance of 10.9 nF cm^{-2}.

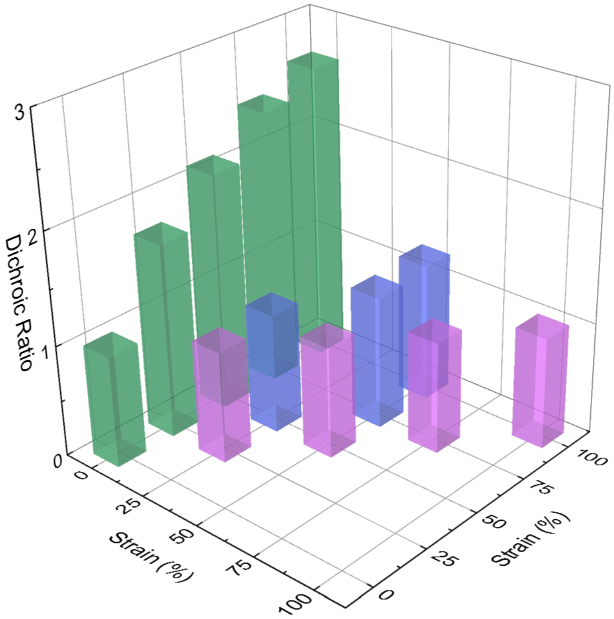

Supplementary Figure S29 | Polymer chain alignment in P4 film under multi-directional strain. The comparison of DR of P4 films under uniaxial or biaxial strain. Conditions of even (e.g. 50×50%, 100×100%, etc.) or uneven (i.e. 25×50%, 50×75%, and 50×100%) biaxial strain are included. Polymer alignment can be observed when the external forces are not equally applied (i.e. uniaxial strain or uneven biaxial strain) through the polymer film.

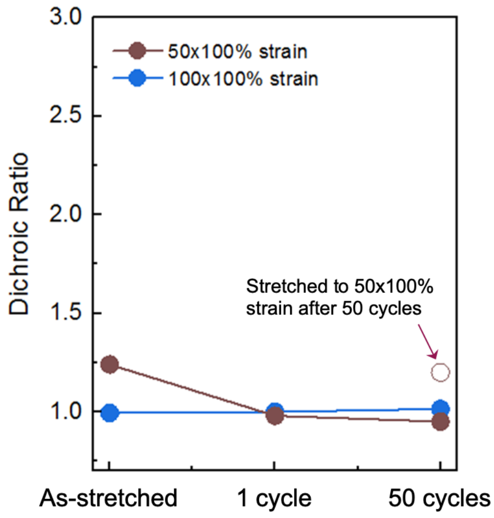

Supplementary Figure S30 | Mechanical stability of P4 film under biaxial strains. The dichroic ratio (DR) of P4 films under 100×100% and 50×100% biaxial strain were explored under multiple stretching/releasing cycles, and the data points were collected at 0% strain after cycling. The DR values of 100×100% stretched films always remain at 1 because polymer chains cannot be aligned to a particular direction under evenly applied stretching force. Films under 50×100% strain, however, show a DR of 1.2 (i.e. polymer chains are aligned to the direction with larger strain (100%)) under strain (both as-stretched and re-stretched after 50 cycles) and of approximately 0.95 (i.e. polymer chains are aligned to the direction with smaller strain (50%) due to the Poisson effect) when released back to 0% strain after cycling, indicating the polymer chains can be slightly aligned to a specific direction when the force is not evenly distributed. One set of samples was measured.

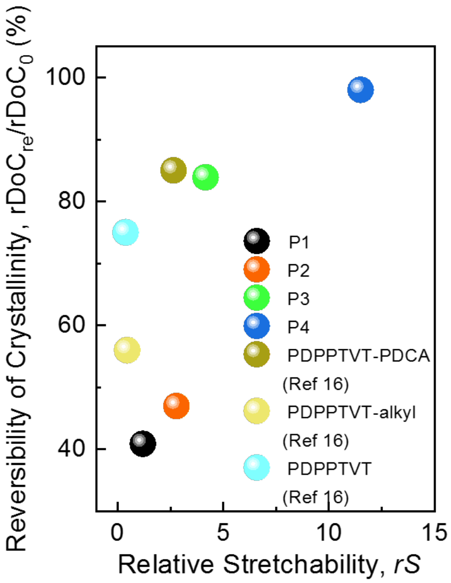

Supplementary Figure S31 | Correlations between reversibility of crystallinity and *rS* values. Reversibility of PSC crystallinity is calculated by rDoC at release state (thin film released from 100 to 0% strain ($rDoC_{re}$) divided by the rDoC at 0% strain ($rDoC_0$)) of the studied and reported PSCs. A general trend shows that the molecular ordering in PSC films has higher reversibility after deformation if the PSC possesses a larger *rS* value.

Supplementary References

S1. Niu, W. et al. Synthesis and properties of soluble fused thiophene diketopyrrolopyrrole-based polymers with tunable molecular weight. *Macromolecules* **51**, 9422-9429 (2018).

S2. Xie, R. et al. Glass transition temperature from the chemical structure of conjugated polymers. *Nat. Commun.* 11, 893 (2020).
